# Organ-On-A-Chip: An Emerging Research Platform

**DOI:** 10.1080/15476278.2023.2278236

**Published:** 2023-11-15

**Authors:** Nithin R, Ayushi Aggarwal, Anne Boyina Sravani, Pooja Mallya, Shaila Lewis

**Affiliations:** Department of Pharmaceutics, Manipal College of Pharmaceutical Sciences, Manipal Academy of Higher Education (MAHE), Manipal, Karnataka, India

**Keywords:** Drug interactions, drug testing, human biology, organ-on-a-chip, toxicity

## Abstract

In drug development, conventional preclinical and clinical testing stages rely on cell cultures and animal experiments, but these methods may fall short of fully representing human biology. To overcome this limitation, the emergence of organ-on-a-chip (OOC) technology has sparked interest as a transformative approach in drug testing research. By closely replicating human organ responses to external signals, OOC devices hold immense potential in revolutionizing drug efficacy and safety predictions. This review focuses on the advancements, applications, and prospects of OOC devices in drug testing. Based on the latest advances in the field of OOC systems and their clinical applications, this review reflects the effectiveness of OOC devices in replacing human volunteers in certain clinical studies. This review underscores the critical role of OOC technology in transforming drug testing methodologies.

## Introduction

Organ-on-a-chip (OOC) devices, referred to as “tissue-chips,” or “micro-physiological systems” (MPS) are microfabricated devices that mimic the properties and physiological functions of complex tissues or specific organs *in vitro*.^1^ These chips provide a potential alternative to the use of animals in research by offering a vehicle to imitate the structural and physiological diversity of human tissues and organ units in a manner that traditional cell culture is incapable.^2^ The OOC makes it possible to create tiny, functional units from one or more organs. These microfluidic cell culture tools offer a more accurate *in vitro* model than petri-dishes in terms of physiology.^3^

A basic functional unit that mimics actual organs has been built using microfluidic devices seeded with human cells and perfused with cell culture medium in a medically realistic way^4^ The optical transparency of the device makes it possible to visualize the whole drug response process at the cellular level that is impossible to achieve in vital organs, which is an additional benefit.^5^ The uniqueness of OOC, along with physiologically relevant readouts opens new possibilities for patient-specific diagnostic and treatment approaches. The purpose of OOC is to simulate crucial functional characteristics of tissues and systems by integrating various fields of study.^6^

The main objective of this review is to highlight the various types of OOC and their applications. The features of the OOC model and the intricacies in designing an OOC are discussed. The review also focuses on how OOC evolved, efforts to mimic critical physiological functions of a human body by the use of sophisticated techniques. The potential functions of the OOC as well as studies conducted by researchers are also discussed. To construct an OOC model of a particular organ, the first step is to identify and isolate the fundamental anatomical components that are necessary for its specific physiological functions, such as nephrons in the kidney, alveoli in the lung, or osteons in the bone.^7^

### Organ-on-a-chip

The title “Organ-on-a-Chip” was first coined in 2010. According to Valverde G et al.^8^ the main objective of using OOC technology is to replicate crucial functional components of organs and tissues by integrating various research fields. The primary idea is to simulate biomechanical stimulation and bodily movement by growing cells in dynamic fluid environments, differentiating them from 3D inert matrices and 2D static culture plates. The organ-on-a-chip (OOC) field has experienced remarkable advancements, propelled by novel techniques that accelerate cell growth and maturation, efficient gene editing, simultaneous testing of multiple samples, and the creation of intricate 3D structures. OOC technology also incorporates primary cells and cells sourced directly from patients. The integration of microchannels allows for precise fluid control within these sophisticated devices, further enhancing their capabilities.

Furthermore, apart from replicating biomimetic 3D structures and cell interactions, studies have shown that mechanical factors such as shear stress and mechanical strain have a notable impact on cell behavior and how they react to drugs. Microfluidics allows for precise control of the microenvironment’s fluid dynamics within a 3D cell culture setup. Consequently, numerous Organ-on-a-Chip (OOC) prototypes use microfluidic technology to recreate the dynamic mechanical conditions on the chip, resulting in improved *in vitro* functional organ models.^9–11^

Some disease devices have been created by using *in vitro* OOC technology which has become the cutting edge of scientific development. This approach goes beyond the conventional 2D-cultures. The technology closely reproduces the microenvironment of tissues in the human body under regulated dynamic settings by displaying the functions not seen in other simpler *in vitro* devices. Because of their physiologically precise readouts, they are used for drug research, preclinical drug testing, and most recently as regulated, small imaging systems that represent certain illnesses.^12^

A crucial problem in the creation of OOC is cell supply. Typically, one cell type is enough to mimic the bare minimum of organ function. For example, a kidney-on-a-chip made entirely of renal tubular cells might not be sufficient to evaluate direct cellular damage. It is sometimes required to utilize two distinct types of cells to produce the settings that truly represent the body or to research how the cells interact with one another. An example of this is the lung-on-a-chip, which includes endothelial cells cultured in the lower channels and alveolar cells in the upper channels of the device.^13^

### Design of OOC devices

Organ-specific microenvironments, serving as fundamental design principles for these devices, are replicated into fine molds. The finished devices can be made from various materials including glass, polymethylmethacrylate (PMMA), polyethylene terephthalate (PET), polystyrene (PS), polyimide (PI), and thermoplastic polyurethane (TPU). Polydimethylsiloxane (PDMS), is a popular choice due to its softness, transparency, biocompatibility, gas permeability, easy replication, and low autofluorescence.^1^

The OOC device was designed to identify the main biological components with distinctive phenotypes, the structural organization of various cell and tissue types, and the mechanical and/or biochemical stimuli running in their immediate environment.^14^ The most popular approach for creating the microdevices that are in these OOC devices has been soft lithography using elastomeric polymers such as PDMS.^15^

These simulations have proven eligible for simulating advanced-level physiological responses and functions that are the outcome of intricate biological interplay between various cell and tissue types. These devices are now highly recommended in research due to their optical transparency, biocompatibility, gas permeability, low cost, and ease of manufacture.^16^

### Significance of OOC devices

Cell-culture techniques, involve growing living cells in a petri dish without blood flow or mechanical stimulation. However, they fail to fully replicate the dynamic environment in actual organs. The absence of mechanical stimulation, including blood flow, in static *in vitro* models such as growing cells in petri dishes represents a significant limitation. These dynamic forces are crucial for simulating the physiological conditions and functional responses of living tissues, and their absence hinders the accurate representation of organ behaviors in traditional cell-culture setups.^17^

OOC technology uses primary human cells over animal cells and has minimal functional components. In addition to using human cells, the ideal strategy will mimic the 3D architecture, environment, and flow properties observed in human organs.

Devices based on microfluidics have been developed to mimic real organs, which are infused with human cells and washed with cell culture fluids in a manner that closely resembles medical settings. This device enables simple flow control, uses minimal samples and reagents, and allows for simultaneous parallel experiments with large numbers of samples.^5^

OOC technology offers numerous benefits that make it a promising approach for modeling human organs and disease. One of the most important benefits of OOC is their ability to replicate essential organ characteristics at the cellular and tissue level, including barrier-like interfaces, complex structures, and interplay of multiple organs. This allows for more accurate and realistic devices of human physiology and disease, which can be used to study disease mechanisms, drug discovery, and personalized medicine.^14^

Another advantage of OOC technology is its versatility in simulating various biological mechanisms. OOC can mimic the effects of mechanical signals such as stretch and flow, which are important in several organs, including the heart, lungs, and blood vessels. It can also sense and control the microfluidic environment of living cells, enabling the study of cell-cell interactions, signaling pathways, and drug responses.^15^

In addition to these benefits, OOC technology can also be used to deliver or stimulate therapeutics in a controlled and targeted manner, making it a useful tool for drug screening and development. Overall, OOC technology has the potential to revolutionize the field of medicine by providing more accurate and predictive devices of human physiology and disease, leading to more effective treatments and personalized medicine.^16^

The OOC has some pros and cons which are described in Table 1.

**Table 1.** Pros and cons of organ-on-a-chip.^18–20^

Pros	Cons
Research speedup in testing the impact of different concentrations of drugs The effect of the drug and the dosage can be examined simultaneously Creates a replicated microenvironment A coin-sized device that is easy to operate, portable, and capable of enormous output	Potential drawback in adsorption of small and hydrophobic moieties Robustness and results of these devices differ from laboratory to laboratory Due to the tiny dimensions of the OOC device, surface effects predominate over volume effects Additional tools may be required to obtain accurate findings

### OOC systems for new drug development

Contrary to traditional cell cultures, OOC technology gives a higher potential for accurate analysis of functional disability, the emergence of harmful outcomes, the pharmacokinetic and toxicity profile, and the potency of medicines.

Micro-physiological tissue devices are being recognized as a valuable resource to improve the transition from preclinical research to clinical studies, and to combat the persistent decline in drug approval rates, despite rising expenditures on research.

Since OOC technology strives to imitate the physical, behavioral, and structural characteristics of tissues and organs it has become a useful option in drug development. After demonstrating a culture media that depicted the connection between the lung and the liver on a rectangular silicone chip, the first paper in 2004 introduced the idea of simulating the natural and biological activities of the human body which uses the cells inside a microfluidic device.^21^

OOC is feasible for studying the effect of diseases on organs due to its organ function mimicking potential.^22^ For instance, in the evaluation of pathogen illness in the kidneys, a standard 2D culture may indicate virus proliferation, but its consequence on renal function cannot be displayed until the 2D culture is matched to an OOC system that could mimic glomerular filtration. OOC can be used to determine a drug’s pharmacokinetic profile and can help in the development and confirmation of medications that specifically target cells and molecules.

OOC technology has shown to be a promising complementary tool to cell cultures and animal devices during preclinical investigations. Conventional experimental devices in drug research and development can be substituted with OOCs since they have exhibited *in vivo*/*in vitro* extrapolation of results.^23,24^

The comparison, advances, and the differences between the OOC and traditional cell culture methods are given in Table 2.

**Table 2.** Comparison of organ-on-a-chip with traditional methods. ^25–27^

Specifications	Organ-on-a-chip	Conventional method
Description	The microfluidics-based cell culture technique thoroughly explains the interplay between cell culture parameters and microenvironmental factors.	Conventional cell culture techniques must be improved in demonstrating cell culture parameters and microenvironmental factors.
Technique employed	Microfluidic technique under-regulated operating conditions at the microenvironmental level will develop cell culture technology even more quickly.	No specific technique
Outcome measure	Provides a unique platform for practical high throughput experiments.	Inaccurate outcomes
Relevance to physiological systems	Offer drug testing devices that are more pertinent to physiological systems.	Inadequate relevance to physiological systems.
Precision	Precise control over the cellular environment	No precise control over the cellular environment
Environment condition	Provide an *in vivo* like dynamic environment.	Fail to produce a dynamic environment.
Accuracy	Provides test results rapidly	No accurate results
Distribution	Diffusion gradient of nutrients and drugs	Equal dispersion of nutrients and drugs

### Lung-on-a-chip (LOC)

Devices of the LOC have shown to be a useful tool for understanding how the immune system reacts to stimuli like cytokines and medications.

The Ingber group first identified LOC which was used as an alveolar-capillary interface model to study the effects of bacteria and inflammatory cytokines on the body. In this study, the impact of silica nanoparticles on epithelial cells was assessed, and the destructive impacts of the nanoparticles, resembled ultrafine airborne particles.^28^

An elastic collagen fiber membrane enclosed within a microfluidic device for testing was fabricated. In this study, human lung microvascular endothelial cells (hLMEs) and primary human alveolar epithelial cells (hAECs) were grown on both sides of the membrane and demonstrated resistance to a 10% strain. Additionally, scientists set permeability across the cell stratum, demonstrating that tiniest particles are carried more accurately. These devices enable scientists to explain the influence of mechanical forces on favorable pharmacokinetics and distribution, potentially improving therapeutic design.^29^

### Lymph node-on-a-chip

Lymph nodes are vital organs of the immune system where efficient and protective immune responses are generated and maintained. Slice cultures of lymph node (LN) tissues preserve cell complexity and 3D architecture while providing easy manipulation of environmental inputs when employed in microfluidic devices. A mechanism to deliver the LN slice inside the upper compartment of PBS (phosphate buffered saline) filled tissue culture was developed. To see how quickly particles move through tissue using a microscope, the device permitted monitoring of particles and molecules in small sections of the slice. Examining diffusion within tissues is crucial because it highlights features of the interstitial space, such as the tortuosity resembling a tissue. Following that, the model was utilized to examine the scattering of 10-kDa dextran in various LN areas. The diffusion coefficient of dextran was shown to be remarkably similar in the paracortex and cortex, indicating that these two regions share a similar extracellular environment.^30^

### Bone marrow-on-a-chip

Multipotent hematopoietic stem and progenitor cells (HSPCs) are the source for all blood cell types. Aleman and his colleagues aimed to generate several roles inside of a model and determined the locations where various cells, including leukemia cells, lymphatic cells, and hematopoietic stem and progenitor cells (HSPCs), travel preferentially. The osteoblastic niche was the most frequent site of migration for leukemia cells, the arterial niche for lymphatic cells, the mesenchymal and sinusoidal areas for HSPCs, and the lymph node niche for lymphoma cells. By examining migration of cells in this regulated environment, researchers were able to develop treatments that prevent some forms of cell migration, such as leukemia and lymphatic cell shifting, but not HSPC movement. Thus, these devices may produce very evident information for both the discovery of new medications and the advancement of biological understanding.^31^

### Microfluidics in cancer immunotherapy and tumor modeling

Microfluidic devices can be used to replicate tumor microenvironments with important physiological characteristics including liquid motion and polymer rigidity, simulating intracellular behaviors and tumor feedback to therapy more precisely.

The investigation of the radiobiological effects of ionizing radiation on the human microvasculature was investigated by mimicking the close structure of small vasculature structures. The systematic comparison between human umbilical vein endothelial cells (HUVECs) cultured within conventional 2D model and the microvasculature-on-a-chip revealed significant differences upon irradiation with X-ray, especially at high doses relevant to stereotactic body radiation therapy (SRBT) and stereotactic radiosurgery (SRS). The structural similarity and qualitative data suggest that such microvasculature chip models are more physiologically relevant than standard 2D cultures. Therefore, better preclinical models of the microvasculature are needed to advance our understanding and to assist in developing new approaches that mitigate their acute and long-term effects on healthy tissues and organs.^32^

Microfluidics switch from 2D to 3D devices has resulted in simulations that more clearly represents the interaction of cell in the tumor microenvironment (TME). Another benefit of utilizing these 3D tumor designs is the capacity to alter the extracellular matrix structure and mechanical characteristics to investigate how different TME factors affect immune cell stimulation and cytotoxicity.

According to a study, OOC systems provide scope to researchers to investigate physio-pathological mechanisms and host immunity in a setting that is very comparable to the individual and the specific illness that affects humans.^22^

An OOC model was developed to demonstrate the pathogenic pathways behind the endothelial alterations seen in pancreatic cancer and identify the chemical mediators of these events.^33,34^

### Intestine-on-a-chip/Gut-on-a-chip (IOC/GOC)

The gut, with its extensive mucosal surface, represents a significant area of interest, leading to the development of several gut-on-a-chip (GOC) devices. Most designs attempt to mimic the cellular organization of the gastrointestinal tract, which comprises an epithelial layer, a mucus stratum, and the extracellular matrix (ECM).^35^

The GOC implemented a device to anticipate and examine the movement of drugs across the biological stratum.^36^ The scientists discovered that, compared to rat tissue, the GOC model enhanced the permeability coefficients of the model agents. The gadget also resembled the villi-like surfaces necessary for material flow within the gut lumen. GOC devices are primarily developed to discover the emergence of attenuated virus infections, usually manifesting as nausea and diarrhea in patients.^37^

A microfluidic device consists of a thin, porous membrane dividing the apical and basal surfaces. In static transwell culture, Caco-2 cells are cultured in a monolayer for approximately three weeks. Static transwell culture refers to the traditional/conventional method of growing cells on a permeable membrane in a static environment, typically within a transwell insert. During this process, the cells are retained in the top chamber on the apical side, where they undergo differentiation into intestinal epithelial cells. In contrast to the static environment of traditional transwell culture, the microfluidic device subjected human colon carcinoma cells to mechanical and moving liquid, which promoted the creation of tight junctions and villi within 6 days, allowing for the investigation of enterovirus infection.^15,38^

The GOC model can be used to study a range of intestinal infections and diseases by concentrating on the impact of mechanical signals such as distortion (peristalsis) or liquid mechanics on infection.^39^ It has three channels, two for perfusion and one for the interstitial tissue space, which contains ECM. In this groundbreaking study, Beaurivage and her team present a pivotal application of a state-of-the-art 3D gut-on-a-chip model as an Organ-on-a-Plate IBD model to delve deeply into the intricate pathophysiology of inflammatory bowel disease (IBD). Their work showcases the remarkable potential of the OrganoPlate platform, replicating critical aspects of inflamed tissue and offering a powerful tool to explore potential therapeutic targets for mitigating the inflammatory responses witnessed in IBD patients. Through meticulous experimentation, the researchers induced inflammatory conditions in the gut-on-a-chip model, yielding remarkable insights. They observed loss of barrier function in the intestinal epithelium, a critical characteristic of IBD. Additionally, the model displayed heightened cytokine production, another pivotal indicator of the disease pathophysiology. Furthermore, the study brought to light the effectiveness of anti-inflammatory compounds in combating the loss of barrier function and effectively reducing cytokine release. In their quest to uncover the underlying mechanisms, the researchers employed on-chip adenoviral siRNA transduction to knock down key inflammatory regulators RELA and MYD88. This targeted approach alleviated the IBD phenotype by reducing cytokine production. The findings of this study demonstrate the potential of the gut-on-a-chip for disease specific modeling, drug discovery and target validation for IBD.^40^

After administration, a drug undergoes absorption, distribution, metabolism, and elimination (ADME) before exerting its effect on the body. The combination of these process yields the pharmacokinetic (PK) and pharmacodynamic (PD) profiles of a drug. Although accurate prediction of PK and PD profiles are essential for drug development, conventional *in vitro* models are limited by their lack of physiological relevance. Recently, microtechnology-based *in vitro* model systems, termed “organ-on-a-chip,” have emerged as a potential solution.

An IOC was fabricated to focus on supplying the microenvironmental components to gut epithelial cells that are lacking in conventional *in vitro* methods. These comprise the peristaltic motion, commensal microorganisms, fluid movement, and 3-D structure of the gut tissue. Caco-2 cells produced more mucus protein MUC-2 when subjected to sufficient fluidic shear stress, which appeared to provide protection against bacterial invasion.^41^

A microfluidic apparatus that can mimic the peristalsis by applying air-powered cyclic vacuum suction was created by Kim et al.^20^ Combining the mechanical strain with fluid flow produced a structure resembling villi and improved Caco-2 cell differentiation. Later, scientists found that the transepithelial morphogen gradient regulates intestinal development using a similar microfluidic system.

### Liver-on-a-chip (LiOC)

LiOC is a 3D *in vitro* hepatic microphysiological system aiming to recreate the conditions of liver tissue on a microscopic scale. LiOC is an advanced throughput system able to simulate hepatocyte conditions and its physicochemical environment.

Recent attempts have been made to imitate the three-dimensional structure of hepatocytes instead of solely cocultivating different cells.^42,43^ Four distinct liver cell types in a double layer were cultured together. The physiologic sequencing involves replicating the cellular organization and interaction of different cell types thereby resembling the physiological structure of the human liver. This approach aimed to mimic the liver’s function contributing to the marked enhancement in liver-specific activities. Improved liver-specific activities were observed after implementing blood circulation and a physiologically sequenced arrangement of various kinds of cells in this liver chip.

Current investigations have prioritized developing numerous devices for liver disease, encompassing conditions such as hepatitis, fatty liver disease, and liver inflammation.^44,45^

A 3D hepatocellular approach was presented for investigating liver diseases, focusing on manipulating the durability, functionality, structure, and polarity of hepatocytes while preserving tissue morphological characteristics, mRNA articulation profile, bile secretion, and greater cell vitality compared to other forms of cell cultures. Significantly, the researchers actively supervised the shaping of the alginate hydrogel that supported the cell culture.^46^

Anti-steatosis compounds were used to produce a 3D culture on a plate using primary essential cells that demonstrated good progress in fatty liver repair. The metabolic processes and adipokine expression observed in this model were reminiscent of those seen in individuals diagnosed with nonalcoholic fatty liver disease.^47,48^

**Figure 1.**
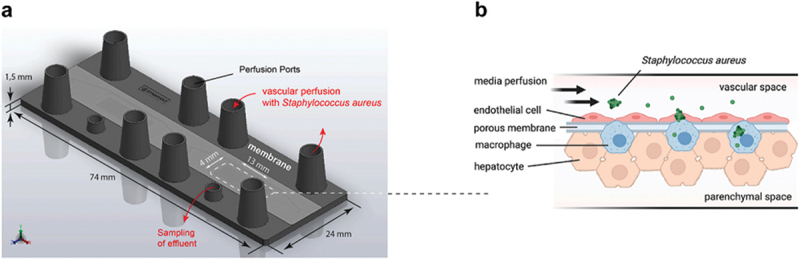
An outline of liver-on-a-chip, reproduced from,^49^ with permission from Elsevier.

A human liver-on-a-chip model, as depicted in Figure 1, was used to show that *Staphylococcus aureus* mainly focuses on macrophages as a crucial specialty that facilitates bacterial survival and phenotypic transition to small colony types (SCTs). According to *in vitro* studies, M2 polarization is a specific activation stage of macrophages associated with SCT growth and larger bacterial loads inside macrophage cells. It leads to increased cell death and diminished capacity to draw circulating monocytes to infection sites. These discoveries added to our understanding of how the macrophages of the liver function and how bacterial persistence is spread when affected by an infection.^49^

**Figure 2.**
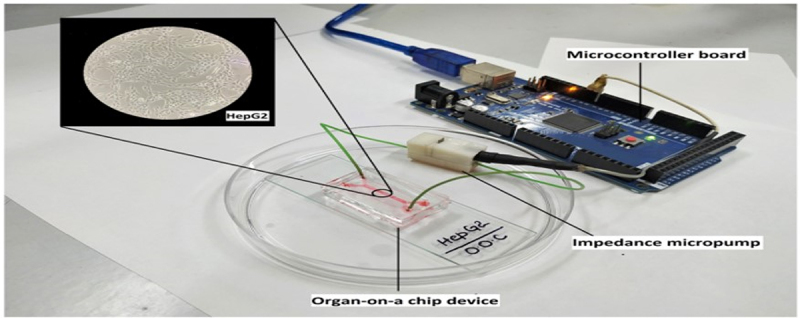
Liver-on-chip containing the Hepg2 cell line, reproduced from^50^ with permission from American Chemical Society.

A free-of-charge resistance micropump (Figure 2) for perpetual nutritional medium delivery in a LiOC containing the Hepg2 cell line was developed (Figure 2). The viability of the cells was confirmed through the observation of healthy cell growth using Hoechst staining and Alamar Blue.^50^

An *in vitro* microdevice, termed as “liver-on-a-chip,” imitates biological lobules of the liver. This microdevice comprises two separate layers: the peak layer with dual seed-feed capabilities and the base layer with culture chambers and diffusion channels. The hexagonal structure and diffusion channels were utilized to replicate the convection-diffusion blood flow in the liver. The chips established demonstrated the formation of a bile-canaliculi network and a three-dimensional tissue-like structure.^51^

### Kidney-on-a-chip

Kidney-on-a-chip model is the combination of a kidney model (ideally a 3D model) with a platform recreating the microenvironment or structure of the kidney *in vivo*. The kidney-on-a-chip project seeks to include proximal tubule cells using a microfluidic apparatus. A multi-layer microfluidic system developed by Jang et al. used to produce primary kidney tubular cells, and the results displayed better polarization of cells and transfer of molecules.^17^ A typical well-based model was known to increase cell polarization, cilia development, albumin distribution, and carbohydrate uptake.^52^

A device that vascularized the proximal tubule on a chip, integrating organoids generated from adult stem cells on a microfluidic chip, and using high-throughput screening was established.^53^

Using a bioprinting technique, Homan KA and his colleagues created 3D human proximal tubules immersed in ECM, which were preserved with vascularization for over two months. The researchers performed confocal imaging to investigate the cellular response to specific inhibitors. In the assay, they observed a unique and significant increase in specific molecular markers associated with tubular function or cellular response pathways upon exposure to the inhibitors. The distinct molecular changes seen in confocal imaging provided valuable insights into the effects of these inhibitors on the 3D proximal tubule model.^54^

A kidney-on-a-chip system aimed at monitoring drug toxicity is an innovative approach developed by Yin and colleagues. It involved cultivating two cell cultures facing each other on membranes, resulting in functional and differentiated kidney tissues. Real-time tracking of cell health and growth was facilitated using fluorescence markers. Jang and his colleagues developed a biomimetic kidney proximal tubule on-a-chip which is an *in vitro* model that can more reliably predict toxicities that can be produced by drugs in humans. They administered the chemotherapeutic drug (cisplatin) and known proximal tubule nephrotoxin via injection into the interstitial compartment of the device. When cisplatin (100 µl) was injected into the interstitial compartment of the microfluidic device or added to the basal chamber of static Transwell cultures, the proximal tubular cells cultured under both conditions exhibited an increase in cell injury as measured by LDH release as well as apoptosis measured by either TUNEL assay or Annexin V staining. However, cells cultured under fluidic conditions appeared healthier at baseline, as indicated by significantly reduced levels of LDH release, indicating a higher toxicity response.^55^ In addition to providing insightful information for preclinical drug screening and toxicity assessments, the study showed the kidney-on-a-chip model’s promise as a trustworthy platform for evaluating drug toxicity in kidney tissues. This kidney-on-a-chip device shows potential for enhancing drug discovery and boosting safer drug testing procedures by precisely simulating physiological settings and enabling controlled monitoring of cellular responses.^56^

### Heart-on-a-chip

Heart-on-a-chip approaches incorporate biological, electrical, mechanical, and topographical cues to facilitate tissue maturation, therefore improving the predictive power for the chamber-specific therapeutic effects targeting adult humans. Several levels of complexity for OOC devices that simulate the human heart have been established. Assessing the contractile and rhythmic functions is the focus of its primary use. As it allows for the connection of the heart with the lungs and liver and detects hazardous effects that can be missing when the medication is tested in an isolated tissue, the preclinical evaluation of a drug may benefit from the OOC application for cardiac reproduction.^57–59^

### Central neural axis, blood brain barrier (BBB)-on-a-chip

The complexity of the human brain makes it challenging to study the central nervous system (CNS) using 2D cultures or animal devices. OOC is feasible to assess if a medicine intended to treat a brain disease may reach its physiological role by creating fully integrated systems that reproduce the membrane features and represent inter individual fluctuation.^22,60^

The model’s ability to include extra cell types, such as pericytes and microglia, expanded its potential in neurovascular investigations and in analyzing how medications affect brain neuronal function. A chip containing neural connections and astrocytes displayed sufficient form, growth, and circulatory role.^61^

### Immune system-on-a-chip

Various microfluidic devices have been created to understand the immune system better and develop new immunotherapies. Devices for immunity in diseases including cancer and inflammatory bowel disease, some devices of the various lymphoid organs such as bone marrow and lymph nodes, and therapeutic devices to test or develop new immune-modulatory medicines have all been used (LN).^62^

OOC systems have biological and therapeutic applications resulting from coordinated cellular activity associated with inflammation that may also evaluate immunity brought by vaccinations or autoimmune illnesses.^62^ Microfluidic devices can access vaccination reactivity that may gather immunoglobulin and would help select immunotherapy candidates. It can also represent the events during the reaction of the immune system to implants.^63^

### Skin-on-a-chip (SOC)

The new advantages offered by organ-on-chip technologies and the necessity of more reliable skin models for drug and cosmetic testing motivated the development of the so-called skin-on-a-chip. These microfluidic devices allow the culture of this tissue under the control of several physical and biochemical parameters such as flows, forces, or chemical gradients. Employing microarray global expression analysis is a valuable method for studying the effect of bacterial illness on the host genomic profile. The skin response to Cutibacterium acnes, *Staphylococcus aureus*, *Staphylococcus epidermidis*, and TLR1/2 agonist was examined in human breast reduction patients using Affymetrix microarray chips. Through Affymetrix HG-133A gene chip microarray analysis, the researchers observed that C. acnes and *S. aureus* increased the synthesis of extracellular matrix components and decreased the markers of cellular differentiation in the genome-wide expression.^64^

The Open-top chip is an innovative technique that enables the precise replication of vital features of tissue functions *in vivo* by managing the microenvironment and recreating the interactions between the epithelium, stroma, and endothelial cells. A robust hydrogel PDMS adhesion was achieved by utilizing a distinct photoactivatable heterobifunctional crosslinker to functionalize the surface of the PDMS. The Open-Top Chip could play a crucial role in the emerging field of organ chips, which are used alongside clinical trials to assess the effectiveness and toxicity of drugs that target the stroma.^34^

**Figure 3.**
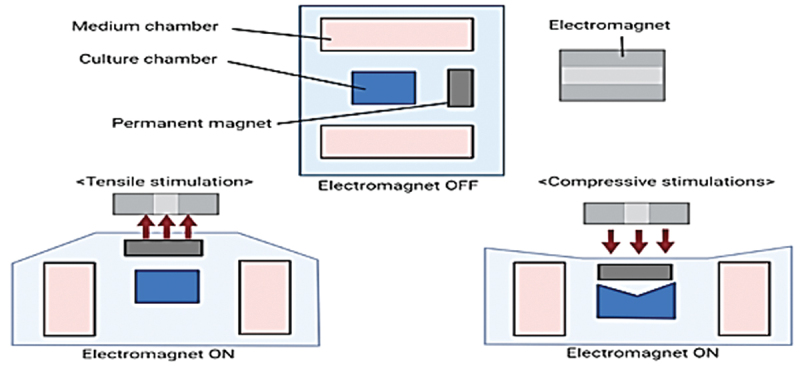
A magnetic stretching skin-on-a-chip.^65^

The skin-on-a-chip model developed by Kyunghee and colleagues, known as the magnetic stretching skin-on-a-chip (MSSC), utilized an integrated electromagnet (Figure 3) to deliver magnetic field-based tensile stimulation. The researchers have investigated the skin’s response to different types of stimuli in MSSC. These stimuli could include mechanical stretching, varying degrees of tension, or other factors mimicking *in vivo* situations. The study findings revealed distinct results for crucial skin proteins, such as involucrin, keratin 10, and filaggrin. Notably, the expression of genes linked to the epidermal barrier, particularly filaggrin, exhibited similar response patterns for both mechanical stretching and other stimuli, indicating the model’s potential to replicate *in vivo* responses. Moreover, the expression patterns of proteins constituting the dermis and epidermis exhibited contrasting reactions to the stimuli, highlighting the MSSC’s capability to simulate specific aspects of the skin’s dynamic behavior under various mechanical influences.^65^

**Figure 4.**
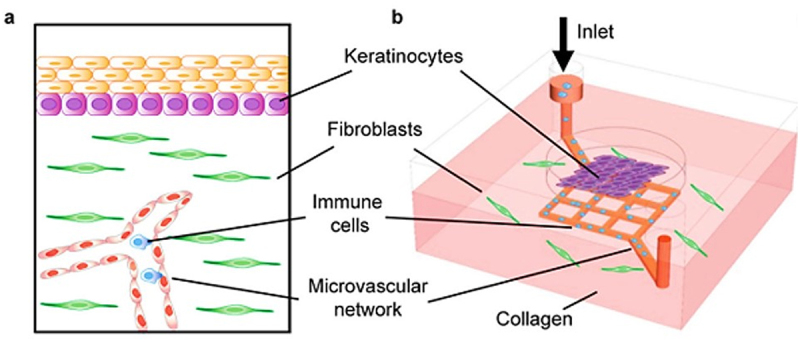
Vascularized skin-on-a-chip, reproduced from^66^ with the permission from springer nature.

A 3D, vascularized skin-on-a-chip (SOC) model that accurately mimics the structure of human skin and allows the perfusion of immune cells and drugs was developed by Sijie et al.^66^ (Figure 4). In this model, the dermis contains endothelialized microvasculature and fibroblasts that respond to various biological stimuli. They include releasing the proinflammatory cytokine IL-8 in response to HSV infection, such as genital herpes in humans. This induction of IL-8 leads to rapid transendothelial extravasation and targeted migration of neutrophils, resembling a significant aspect of the morphological and pathophysiological response to HSV infection. The vascularized SOC model provides an appropriate platform for replicating natural HSV infection and testing potential treatments before proceeding to clinical trials, thus reducing animal testing and offering insights into how the skin responds to specific biological stimuli during infections like HSV.

### Eye-on-a-chip

Ophthalmic drug discovery has received critical attention in recent years and *in vitro* physiologically representative models undoubtedly speed up the preliminary drug screening. Zitong Yu et al., developed a human cornea-on-a-chip using human corneal cells and a collagen-coated porous membrane to replicate the structure of the cornea. This microfluidic device includes separate channels for different cell types (Bowman’s layer, stroma, and Descemet’s membrane), enabling us to create a fully integrated human cornea on the chip. They utilized this setup to assess the effectiveness of extracellular vesicles (EVs) in repairing corneal epithelial wounds. The porous membrane divides the upper and lower channels, allowing for distinct fluidic access and control of environmental conditions. Additionally, the upper layer of the device, made from polydimethylsiloxane (PDMS), creates an air-liquid interface (ALI) for corneal epithelial cell culture. In their study, the scientists employed a cell-free approach with mesenchymal stem cell-derived extracellular vesicles (MSC-EVs) to assess the barrier effects of the cornea-on-a-chip. MSC-EVs, small membrane-bound vesicles released by mesenchymal stem cells (MSCs), carry various bioactive molecules that mediate essential cell-to-cell communication and tissue repair processes. The researchers have validated the effectiveness of MSC-EVs in treating scratch wounds and discovered that MSCs from bone marrow can produce EVs that expedite the healing of mild corneal wounds that accurately represents the specific biological phenotype of the human cornea.^67^

In this organ-on-a-chip model of the outer blood retinal barrier (oBRB), the coculture of retinal pigment epithelium (RPE) and human umbilical vein endothelial cells (HUVECs) within a microfluidic chip with a polyester membrane separating the microchannel from the open-top culture chamber allows for the reproduction of a functional aspect of the tissue/organ. A collagen hydrogel within the microchannel and a subtractive micropatterning technique to create a well-defined microvessel shape enable the emulation of the physiological interactions and barrier properties of the outer blood-retinal barrier. This functional aspect of the tissue/organ in the model facilitates the study of drug transport, disease mechanisms, and potential therapeutic interventions relevant to the outer blood retinal barrier function in the eye.^12^

**Figure 5.**
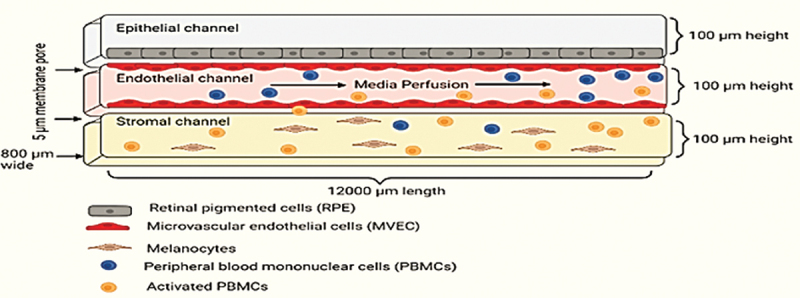
Choroid-on-a-chip depicting the choroid layer of the eye. ^24^

Madalena and colleagues^24^ developed an advanced *in vitro* model of the choroid layer of the eye called the choroid-on-a-Chip (COC) shown in **Figure 5**. This model incorporates human melanocytes and microvascular endothelial cells and is coated with retinal pigmented epithelial cells. The COC model exhibits immunocompetence by using a peripheral immune cell perfusion system. The researchers demonstrated regulated immune cell recruitment into the stromal compartments using a vascular monolayer and cytokine release patterns that mimic *in vivo* conditions. Additionally, they provided a detailed description of this innovative model and its response to two different antigens: (i) T-cell bispecific antibody (TCB) containing the T-cell receptor binding domain and (ii) cyclosporine, an immunosuppressive medication, when T-cells were activated.

### Body-on-a-chip

OOCs can be used to create many tissues mimicking the meticulous process of the human body, and to identify the pharmacokinetic and pharmacodynamic profiles of drugs.^68^ A heart-liver-skin device was created for the comprehensive analysis of drugs applied topically and for the assessment of acute and systemic toxicity.^69^

A multi-tissue system containing organoids constructed entirely with human cells and tissues from the brain, liver, lung, heart, and endothelium proved prodrug transformation and metabolizing capabilities as well as displayed cardiac toxicity.^31^ This type of device has the potential to be employed for pharmacokinetic profiling of pharmacological drugs, as demonstrated by the research on acetaminophen ADME using a microphysiological system.^23^

### Multiorgan-on-a-chip

A physically realized statistical, metabolic pathway PK/PD (pharmacokinetics and pharmacodynamics) model was constructed using macroscopic level perfusion fermenters interconnected by fluidic ducts. This setup enabled organ crosstalk, akin to how blood circulation physiology connects organs within the human body and facilitated a more comprehensive understanding of drug absorption, distribution, metabolism, and excretion (ADME) in the context of the interconnected organ-on-a-chip model.^70,71^

A microscale cell culture analog of three organ chambers interconnected by fluidic connections was developed.^72^ To comprehend the experimental outcomes from the multiorgan-on-a-chip system, Sung JH and his colleagues employed an integrated chip-based approach, incorporating 3D hydrogel cell culture and pharmacokinetics and pharmacodynamics (PK/PD) concepts. Applying PK/PD concepts allows for a deeper insight into how drugs are absorbed, distributed, metabolized, and exert their effects on different organs within the interconnected chip-based system, thus providing valuable information on drug behavior and efficacy. This integrated approach enhances our understanding of drug responses and interactions within the complex organ-on-a-chip environment, paving the way for more effective drug development and personalized medicine.^73,74^

### Design of multi organ-on-a-chip

Multiorgan-on-a-chip has the ability to physiologically replicate the human body. The viewpoint contends that the various chip design features may impact the outcomes of chip studies.^75^

Vinci B et al.^76, 77^ described numerous physiological characteristics, including metabolic activity, pulse activity, blood circulation activity, and organ capacities that may be described by a power law in accordance with the allometric scaling law.

$$P=a\Delta M^{b}$$

P is a physiological parameter, M is an organism’s mass, and the connecting parameters a and b are allometric values. It makes sense that the scaling laws rule might be applied to the microscale, that is, used to predict ideal organ dimensions and crucial process variables of the “microscale organism” chip, given that a multi organ-on-a-chip is basically a microscale human being. Multiorgan systems with different configurations of liver cells, endothelial cells, and fatty tissue cells were created using the allometric scaling law.

To develop invariable constraints for creating several OOC, Abaci and his colleagues^78^ presented a more complicated method derived from previous methods. The first of these two steps in this strategy involves using an OOC to achieve steady-state levels of intrinsic bioactive compounds such as glucose, oxygen, and amino acids that mimic the absolute values in the body of the individual to assess the parameter set for a human physiologically based pharmacokinetic model (PB/PK). In the second step, where individual PK needs to be replicated, “human-on-a-chip” (HOC) was utilized.

### Mutiorgan-on-a-chip for pharmacokinetic/toxicokinetic reduction

An “Integrated insert in a dynamic microfluidic platform” which is a 96-well microplate, can replicate the presystemic elimination through the connected breeding of metabolic organs and hepatocytes. The device’s unique feature is the ability to insert cell culture plugs into the microchip.^56,79–81^

The effectiveness or lethality of medications and their byproducts was evaluated in specific organs are linked to the stomach, liver, or both.^82–84^ An attempt was made to evaluate antitumour medications in a multiorgan-on-a-chip containing the liver. To assess uptake and metabolic activity, the lethal reaction to acetaminophen, mouth absorption, and the adverse reaction to nanoparticles, the absorption step was introduced to the GI tract module. This idea has indeed been developed into a more effective, customer friendly microfluidic device with gradient induced flow to evaluate the toxicity and presystemic elimination of anticancer medicines.^59,85^

Instead of using standard cell lines, Kuhul J and their team^86^ are going toward using primary cells, induced pluripotent stem cells, spheroids, and organoids in order to study human tissues and cell interactions. They are creating a chip called the “HUMIMIC Chip2” that has a skin device and liver spheroids to test how drugs affect the skin and liver. For further research they planned to study how the drugs are broken down and how the cells in the skin and liver work together. They are also working on adding more organs to the chip, like the gut, heart, lungs, and fatty tissues.

To demonstrate multiorgan toxicity, a 4-organ system comprising heart, muscular, brain, and hepatic samples was fabricated. Drug-induced cardiotoxicity and hepatotoxicity were observed, and measurements of functional indicators for each tissue were taken.^59^

### Multiorgan-on-a-chip for pharmacokinetics and toxicological aspects

Multiorgan-on-a-chip is a potentially ideal method for modeling how organs interact as diseases progress. It is a networked system with several micro physiological systems.^87^ It enables the connection of cocultures of various cell types and the realization of the intricate, dynamic, and sometimes unpredictable interplay between numerous organs.^88^

### Advanced human BBB-on-a-chip: a new platform for Alzheimer’s disease studies

B.M. Maoz and colleagues integrated various brain cells into microfluidic organ chips, enabling them to investigate the PK/PD parameters and biochemical interactions between blood brain barrier (BBB) cells and neurons. With these platforms with different brain cell types, they demonstrated the metabolic relationship between BBB cells and neurons. The multi-chip system effectively emulated the human neurovascular unit (NVU), allowing detailed analysis of individual cell type contributions to NVU functions. This comprehensive approach becomes particularly relevant for understanding and addressing neurological disorders such as Alzheimer’s disease, where the NVU plays a pivotal role.^7^

Microfluidic organ chips represent a cutting-edge tool for investigating the neurovascular unit (NVU) in Alzheimer’s disease. These chips allow researchers to study the complex interactions between brain cells that are central to the disease. By simulating drug effects and exploring cell interactions within the NVU, these microfluidic models provide valuable insights into the underlying mechanisms of Alzheimer’s pathology. Understanding how different cell types influence drug transport, efficacy, and toxicity is crucial for developing targeted therapies. Moreover, coupling three chips (body-on-chip, human-on-chip, and multiorgan-on-chip) to model influx across the blood brain barrier (BBB), brain parenchymal compartment, and efflux across the BBB provides a more realistic representation of drug effects in the brain. Three chips are used to investigate the NVU in Alzheimer’s disease, representing the blood brain barrier, brain tissue, and efflux transporters to study drug effects and interactions within the NVU. This enables researchers to examine how drugs interact with the NVU, paving the way for the development of potential treatments for Alzheimer’s and other neurodegenerative diseases. In summary, microfluidic organ chips offer a promising avenue for studying the NVU and advancing our understanding of Alzheimer’s disease. These models play a vital role in exploring disease mechanisms, identifying potential therapeutic targets, and ultimately developing effective treatments for this complex and devastating disorder.^7^

An advanced human BBB model that is capable of simulating both healthy and pathological astrocytes, thereby creating a more physiological system has been developed. This BBB-on-a-chip model features a 3D astrocytic culture with polarization and aquaporin4 (APQ4) expression, which promotes astrocytic end-feet processes and reduces the expression of the reactive astrocyte marker lipocalin2. In contrast to the previous 2D astrocytic culture BBB model, the new system provides a more physiologically relevant environment that allows for the validation of receptor-mediated transcytosis (RMT) of nanoparticles in a mechanistic manner, as well as accurate qualitative and quantitative assessment of nanoparticle distribution. This novel BBB-on-a-chip device provides a platform to investigate receptor-mediated transcytosis (RMT) of nanoparticles in a mechanistic manner. It allows accurate qualitative and quantitative assessment of nanoparticle distribution, which is relevant in understanding drug delivery across the blood-brain barrier in Alzheimer’s disease treatment.^89^

Moreover, the BBB-on-a-chip model has been instrumental in studying the RMT pathway. The gene expression of RMT transporter proteins, tight junction proteins, and solute carrier family proteins was higher in the microfluidic device with pericytes and astrocytes, creating a more complex 3D environment. The superior maturation and reactivity of astrocytes in this model as perivascular cells demonstrate its potential relevance for Alzheimer’s disease, where astrocytes play a crucial role in maintaining the brain’s microenvironment and supporting neuronal function. In summary, the BBB-on-a-chip model developed by Ahn SI. It provides a more physiologically relevant environment to study the interactions and transport mechanisms involved in Alzheimer’s disease. The ability to simulate healthy and pathological astrocytes and analyze RMT pathways enhances our understanding of drug delivery to the brain. It holds promise for advancing research in Alzheimer’s therapeutics and treatment strategies.^89^

BBB-on-a-chip devices have been verified to be valuable in RMT pathway studies. The gene expression of RMT transporter proteins, such as P-gp, LRP1, and AGER, tight junction proteins, and solute carrier family proteins, such as GLUT1, was found to be higher in the microfluidic device with pericytes and astrocytes, compared to the identical model without them. In a more complex 3D environment, astrocytes demonstrated superior maturation and reactivity as perivascular cells in the BBB-on-a-chip model.^89^

### Brain-liver-on-a-chip

A micro physiological system (MPS) integrates a confined microfluidic device containing a BBB model produced from human induced pluripotent stem cells (hiPSCs), together with a cerebral brain and liver spheroid model derived from the same donor. Both metabolism and BBB permeability were evaluated using the two model drugs, propranolol, and atenolol. Both compounds had a penetration behavior similar to that *in vivo* and underwent *in vitro* metabolism.^90^

### Gut-liver-on-a-chip

A gut-liver-on-a-chip system with Caco2 cells in coculture with HT29 cells in the intestinal compartment, and single donor primary hepatocytes in the hepatic compartment were used to investigate intestinal permeability, metabolism (intestinal and hepatic), and potential interactions between these processes. Mycophenolate mofetil, a prodrug, was examined for a quantitative assessment of the gut-liver connection. Mechanistic modeling of experimental data was utilized to estimate the clearance and permeability characteristics of the prodrug, active drug, and glucuronide metabolite. Results from this study demonstrated that the gut-liver-on-a-chip. It can simulate the metabolism of mycophenolate mofetil in both the intestine and liver, suggesting that it could predict the pharmacokinetics and drug-drug interaction risk of other orally administered drugs that exhibit complex *in vivo* behavior.^91^

### Kidney-liver-on-a-chip

A model of kidney damage using hydrogen peroxide and human kidney and liver organoids combined through microfluidic channels to maintain physiological functionality was developed by Vivian and his colleagues. Notably, additional microfluidic channels made from human MSCs have shown the ability to accelerate the repair of renal tubuloids following oxidative injury by promoting the restoration of barrier integrity and functional transport. The MOC model replicates findings from animal devices regarding the biodistribution and therapeutic efficacy of mesenchymal stromal cell-derived small extracellular vesicles (MSC-sEVs). Its human background enables a comprehensive investigation of the mechanism of action (MOA) and the identification of potential adverse effects.^92^

### Skin-liver-organ-on-a-chip

Researchers have investigated the potential of a topical approach using the HUMIMIC Chip2, the first multiorgan chip technology to incorporate skin devices. This model can assess the impact of various exposure scenarios on the pharmacokinetics and pharmacodynamics of two topically applied compounds, hyperforin and permethrin. The results revealed that repeated applications of the substances resulted in higher concentrations of parent chemicals and metabolites compared to a single application. Furthermore, the application route affected specific compounds frequency and gene induction. The model demonstrated excellent repeatability within and between labs, highlighting its usefulness in providing relevant information about the potential *in vivo* effects.^86^

## Conclusion

Organ-on-a-chip (OOC) technology can be a suitable alternative to clinical and preclinical studies. Incorporating cellular technology has significantly enhanced the digital resolution of drug screening data compared to traditional/conventional cell culture methods. A vital advantage of this technology is the ability to identify drug interactions with multiple organs using multiple chips. OOCs can help us determine why some side effects appear during clinical trials that were not seen in animal testing. Organ-on-a-chip devices can personalize cancer treatment by mimicking how a specific patient’s cancer will react to therapy. In future, we can create a tumor model in an organ chip and see how the cancer cells move through the blood vessels, survive, and develop new tumors in other parts of the body. However, it is not difficult to foresee that organ-on-a-chip technology will play an instrumental role in the development of nanotherapeutics in the future.

In addition to providing insightful information for preclinical drug screening and toxicity assessments, the study showed the kidney-on-a-chip model’s promise as a trustworthy platform for evaluating drug toxicity in kidney tissues. This kidney-on-a-chip device shows potential for enhancing drug discovery and boosting safer drug testing procedures by precisely simulating physiological settings and enabling controlled monitoring of cellular responses.

Currently, most organ-on-a-chip devices focus on one or a few organs, but connecting them to create a single chip that imitates all significant organs is crucial. This requires considering interorgan scaling, expected flow rate, media, and interrelated functionalities. While some considerable organs are simulated using this technology, there needs to be more research on other organs such as adipose tissue, retina, and placenta. The ultimate goal is to develop a “body on a chip” technology.
